# Age‐related aspects of human IgM^+^ B cell heterogeneity

**DOI:** 10.1111/nyas.12823

**Published:** 2015-07-07

**Authors:** Victoria Martin, Yu‐Chang Wu, David Kipling, Deborah K. Dunn‐Walters

**Affiliations:** ^1^ The Feinstein Institute for Medical Research, ManhassetNew York; ^2^ Stanford University School of Medicine, StanfordCalifornia; ^1^ Division of InfectionImmunity and Inflammatory Disease; ^2^ Randall Division of Cell and Molecular BiophysicsKing's College London Faculty of Life Sciences and MedicineGuy's CampusLondonUnited Kingdom; ^3^ Institute of Cancer and GeneticsSchool of Medicine, Cardiff UniversityHeath ParkCardiffUnited Kingdom

**Keywords:** IgM memory, immunoglobulin repertoire, aging

## Abstract

The CD27^+^IgD^+^ B cell population, known as IgM memory, reduces with age. It is thought that this population is responsible for pneumococcal polysaccharide T‐independent responses, and that the age‐related reduction might be partially responsible for the increased susceptibility of older people to bacterial pathogens. There are other IgM^+^ B cell populations that do not express IgD. We compared the different IgM populations using high‐throughput sequencing of the immunoglobulin (Ig) gene repertoire and multidimensional cell phenotyping and found that the different populations of IgM cells, defined by CD27 and IgD expression, have repertoire differences. Some of these differences are likely indicative of different selection pressures in an immune response, although the older individuals were found to have a changed repertoire in naive B cells, which may contribute to some of the changes seen in memory cells. In addition, even within the CD27^+^IgD^+^ IgM memory population there are multiple cell types. We show that the level of IgM expression varies substantially and hypothesize that this distinguishes between T‐dependent and T‐independent types of IgM memory cells. Significant age‐related changes in the relative proportions of these populations may exacerbate the reduction in T‐independent responders in old age.

## Introduction

The origin and function of the CD19^+^CD27^+^IgD^+^ B cell population known as IgM memory is still a subject of some controversy.  The term *IgM memory* was originally given to these cells, as they have mutations in their immunoglobulin (Ig) genes and express CD27.^1^, ^2^ In humans, it is thought that they are the recirculating equivalent of the marginal zone cells of the spleen, where IgM cells containing mutations are also found.^3^, ^4^, ^5^ Some believe they are the B cells that respond to T‐independent stimuli,^4^, ^5^, ^6^, ^11^ while others argue that they are precursors to switched memory cells in a T‐dependent response.^7^ More recently, it has been proposed that this population contains the human equivalent of mouse B1 cells.^8^ In our lab, we have shown that the Ig gene repertoire of IgM memory cells differs markedly from that of switched memory cells,^9^ and therefore we would argue that the majority of the population would respond to different stimuli than the switched cells in order for this difference to appear. However, there is no denying the evidence that some IgM and IgG cells can originate from the same B cell precursor, presumably in the same reaction,^7^ nor that persistent IgM memory cells can be formed in a T‐dependent response, at least in mice.^10^

Both the putative human B1 cell population and the IgM memory population have been shown by some groups to decrease with age.^11^, ^12^ Since IgM memory is thought to provide protection against encapsulated bacteria, it could be argued that it is this decrease that causes the increased risk of morbidity and mortality due to pneumonococcal pneumonia in older people.^11^ Indeed, for many years the poor functionality of older serum against pneumococci (as measured by the opsonophagocytic assay) was puzzling in face of the fact that these patients had the same levels of IgG as younger vaccine recipients. However, Park and Nahm showed that removing IgM from the serum can decrease serum functionality.^13^ In the same year, we showed that the antipneumococcal IgG titer was the same in older people, but that IgM and IgA were deficient.^14^

In light of the heterogeneity and proposed function of these IgD^+^CD27^+^ cells, the name “IgM memory” is perhaps confusing.  There are also other IgM‐expressing cells that are not naive but have lost IgD and may or may not express CD27.  The differences between switched memory cells that differ in expression of CD27 have been discussed elsewhere,^15^ and it is important to note that the CD27^–^ memory population increases with age^16^ and with autoimmunity and chronic viral challenge.^16^, ^17^, ^18^ During B cell development there are formative events that increase the representation of certain types of Ig genes, by expansion in response to challenge, and events that decrease the use of some Ig genes, by deletion as a result of autoreactivity. Ig gene repertoire analysis can be used to infer whether the formative events for a particular B cell population are different from those of another population.  Ig heavy chain genes are formed by random recombination of variable (*IGHV*), diversity (*IGHD*) and joining (*IGHJ*) genes, with additional diversity gained by the imprecise joining of the segments at the V–D and D–J junctions, whereby nucleotides are inserted or deleted into these junctions by terminal deoxynucleotidyl transferase. The junctional region is known as the heavy chain complementarity determining region 3 (*CDR‐H3*) and can be used as a fingerprint to identify copies of a particular Ig gene rearrangement from the same lineage.  The B cell repertoire can be described in terms of the frequency of use of the different types of genes.

In this paper, we sorted cells into different subsets based on CD27, IgD, and CD10 staining and used IgM constant region‐specific primers to produce a large number of *IGH* sequences from individuals aged from 21 to 87 years old. We describe the different populations of antigen‐experienced IgM cells in relation to their Ig gene repertoire and demonstrate the changes with age at a point 28 days after vaccination with influenza and pneumococcal polysaccharide vaccines.  Additionally, we used a large panel of markers, using mass cytometry, and we show the heterogeneity of IgM memory cells with respect to different levels of IgM expression and identify two distinct populations whose frequencies are altered in aging.

## Methods

### B cell isolation and cell sorting

Peripheral blood mononuclear cells (PBMCs) were isolated from a total of 14 young (21–45 years) and 16 old (62–87 years) healthy volunteers. Written consent was obtained in accordance with the Declaration of Helsinki after approval from the Guy's Hospital research ethics committee (REC 08/H0804/57 and 09/H0504/39). PBMCs were isolated using Ficoll plaque Plus (GE Healthcare) and Leucosep tubes (Grenier Bio‐One Ltd).

**Figure 1:**
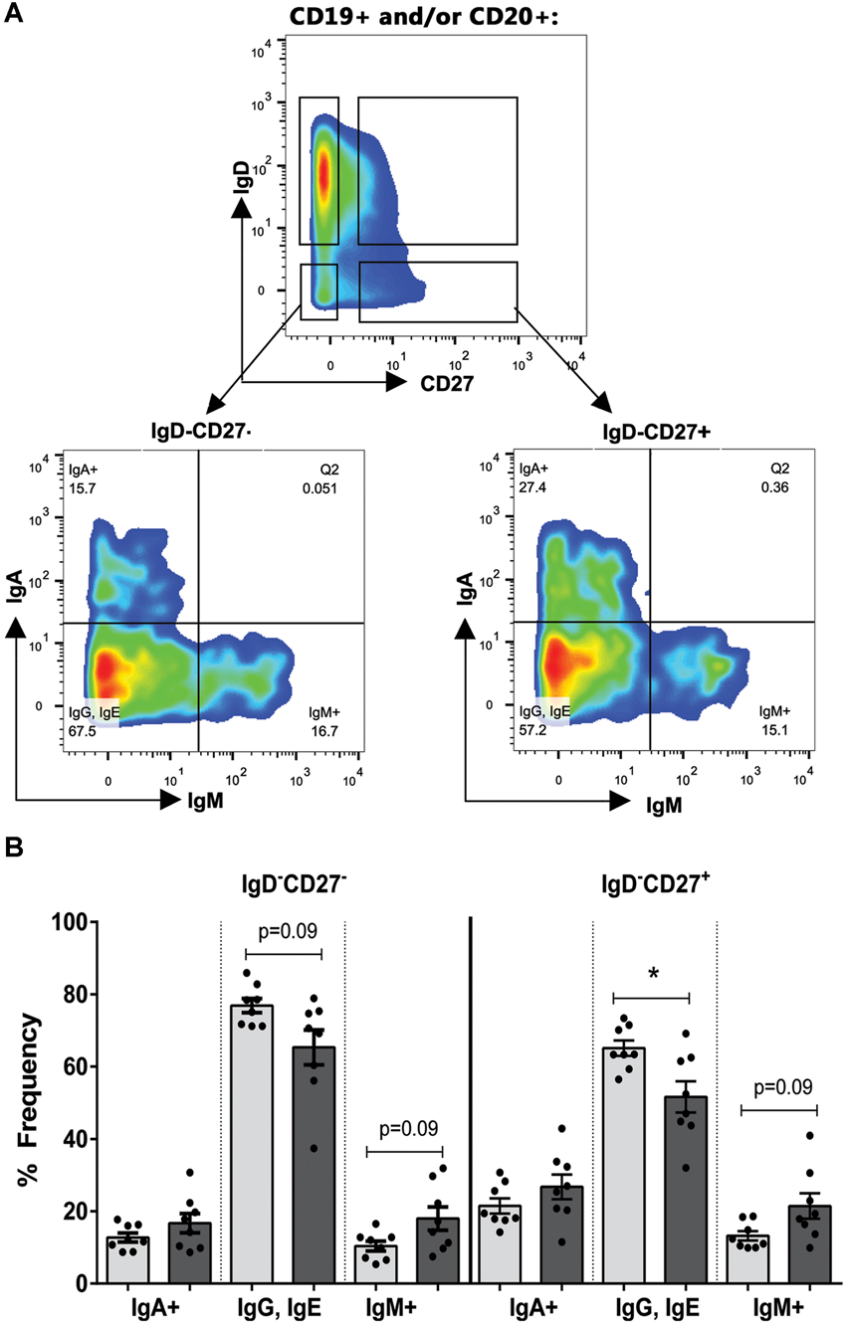
Age‐related changes in B cell populations. (A) Gating used to identify populations of naive (IgD^+^CD27^–^), IgM memory (IgD^+^CD27^+^), IgD^–^CD27^+^, and IgD^–^CD27^–^ populations from pre‐gated CD19^+^ and/or CD20^+^ B cells using metal‐tagged antibodies and mass cytometry. Further gating of IgD^–^ populations into IgA^+^, IgM^+^, and IgA^–^IgM^–^ cells (referred to as IgG, IgE). (B) Quantification of subclasses of cells within the IgD^–^ populations in young and old donors. *n* = 8 young (light gray) and *n* = 8 old donors (dark gray), *denotes *P* < 0.05 by Mann–Whitney U test, bars are standard error of the mean (SEM).

For high‐throughput sequencing analysis, CD19^+^ B cells were positively selected for using the MACS B cell Isolation Kit (Miltenyi Biotec), stained with CD10‐APC, CD27‐FITC (Miltenyi Biotec) and IgD‐PE (BD Bioscience PharMingen) at 4 °C (15 min) and analyzed on a FACSAria (BD Biosciences PharMingen). Five subsets were separately collected (Fig. 1), as previously published: transitional (IgD^+^CD27^–^CD10^+^), naive (IgD^+^CD27^–^CD10^–^), IgM memory (IgD^+^CD27^+^), IgD^−^CD27^+^, and IgD^–^CD27^–^ into 180 μL of Sort‐Lysis RT buffer (SLyRT).^9^ SLyRT comprises 150 ng/μL pd(N)_6_ (Invitrogen), 2.5 U/μL RNAse inhibitor (Bioline), 0.13% Triton X‐100 (Sigma‐Aldrich), 12.5 mM DTT, and 500 μM each deoxyribonucleotide triphosphate (dNTP) mix (Promega) in 1× First‐Strand RT buffer (Invitrogen) final concentration (i.e., in 200 μL). IgM‐specific PCR primers were later used to ensure that all sequences came from IgM^+^ cells, and the IgM‐sequenced IgD^–^ cells are henceforth referred to as IgM‐only CD27^+^ or IgM‐only CD27^–^ cells.

### Mass cytometry and data analysis

For mass cytometry experiments, PBMCs were stained with CD14‐FITC and CD3‐APC (Biolegend), and a population of enriched B cells (CD3^–^CD14^–^) was collected into 50% FCS (Biosera) and 50% RPMI‐1640 (Gibco). The CD3^−^CD14^−^ enriched B cells were labeled with a rhodium intercalator (Rh103, DVS Sciences) followed by intracellular and extracellular staining with a panel of 30 different metal‐tagged antibodies (DVS Sciences, BD Biosciences and Biolegend). Cells were fixed, iridium stained (Ir193, DVS Sciences), and normalization beads (DVS Sciences) were added before analysis on a CyTOF (DVS Sciences). Between 1 and 5× 10^5^ stained cells were analyzed per sample.

Data were normalized and files were concatenated and cleaned up to remove debris (by gating on cell length and DNA^+^ cells), to exclude normalization beads (Ce140^–^ cells), to positively select intact cells (Ir191^+^ Ir193^+^), to positively select live cells (Rh103^–^Ir193^+^), and to identify CD19^+^ and/or CD20^+^ B cells. Within FlowJo v10 (TreeStar), conservative IgD and CD27 gating was employed to identify the four distinct B cell populations (naive IgD^+^CD27^–^, IgM memory IgD^+^CD27^+^, IgD^–^CD27^+^, and IgD^–^CD27^–^). The abundance of different classes of B cells (IgM^+^, IgA^+^, and IgM^−^IgA^−^) was quantified and compared between different IgD^−^ cell populations. To phenotypically characterize and compare the different cell subpopulations and samples, histograms of the mean fluorescence index (MFI) or frequency of positive cells by expression of different markers were overlaid and statistical comparisons made using Student's *t*‐tests, the Mann–Whitney U test, or 2‐way ANOVAs.

### High‐throughput sequencing and data analysis

cDNA synthesis was performed by adding 500 U SuperScript III reverse transcriptase (Invitrogen) to the 180 μL of SLyRT buffer containing the sorted cells. The following RT reaction was performed: 42 °C (10 min), 25 °C (10 min), 50 °C (60 min), and 72 °C (15 min). The Ig genes were amplified as in Ref. 9. Briefly, Ig genes were amplified using a semi‐nested PCR with isotype‐specific primers. A 25 μL PCR1 reaction containing 6.25 μL of cDNA, 0.625U Phusion DNA polymerase (NEB), 200 μM each dNTP, 41.75 nM each of 5′ *IGHV* gene family primer, and 250 nM IgM‐specific constant‐region primer, was run at 98 °C (30 s), 15 cycles of 98 °C (10 s); 58 °C (15 s); 72 °C (30 s), and 1 cycle of 72 °C (5 min). PCR2 was then performed using 2 μL of PCR1 product, 0.5 U Phusion DNA polymerase, 200 μM each dNTPs, 41.75 nM each of 5′ *IGHV* gene family, and 250 nM nested constant‐region primer where all primers contained matched multiplex identifiers (MIDS), at 20 (PCR2) cycles of 98 °C (10 s); 58 °C (15 s); 72 °C (30 s), and 1 cycle of 72 °C (5 min). PCR products were purified and sequencing was carried out on the Roche 454 Titanium platform by LGC Genomics (Germany).

Downstream data clean‐up and processing were carried out as previously published.^9^ The data were first subjected to a stringent set of rules to remove sequences with two different MIDs or internal MID sequences and to remove sequences that were biologically implausible (i.e., containing multiple V or J genes, a C‐terminus at both ends, or an internal subclass motif and a 3′ subclass primer). Finally, short sequences were removed from the data where the C to *IGHV* regions are smaller than the following: Cα < 289 nucleotides, Cμ < 333 nucleotides and Cγ < 409 nucleotides. Approximately 50% of sequences pass this quality control step.

After the initial quality control, sequences underwent Ig genotyping as follows. Ig gene usage and the CDR‐H3 junction regions were determined using V‐QUEST.^19^ ProtParam was used to determine the physicochemical properties of the CDR‐H3 peptide between the conserved first (cysteine) and last amino acid (tryptophan).^20^ Clonotype clustering was carried out on CDR‐H3 using a clustering matrix such that sequences with the same *IGHV* gene and within 10 nucleotides in length of the test sequence were used to make a distance matrix, which was then used for hierarchical clustering. Related sequence pairs were determined as having a distance of 0.25.^9^ Once the clusters of related sequences were established, the modal sequence was determined to be used as a representative of this group and was assigned as a unique sequence. Only the unique sequences were used within this analysis to remove any skewing that could have arisen from PCR amplification. Annotated and cleaned data were combined and subsequent analyses performed in Excel (Microsoft). Owing to the difficulties of accurately studying any differences caused by mutation, and the focus on the IgM sequences, we have made no analysis of somatic hypermutation.

## Results

### Age‐related changes in B cell populations

Reports on the frequency of IgM memory cells show either no change^16^ or a decrease^11^ with age. The frequency of CD27^–^ memory cells has been shown to increase with age.^16^   However, data on the proportions of IgM‐only cells in the IgD^–^ populations are scarce.  We pregated for CD19^+^ and/or CD20^+^ cells and quantified the proportions of IgM and IgA cells that are IgD^–^ in eight young (18–32 years) and eight old (62–82 years) individuals using mass cytometry.  Since we could not use anti‐IgG in our staining panel, we assumed that the CD19^+^ and/or CD20^+^ B cells that did not stain with IgM or IgA were mainly IgG^+^ with possibly some expression of IgE (here referred to as IgG, IgE (Fig. 1A)). It is worth noting that, for mass cytometry experiments, the negative cells have no mass and so are found along the axis, meaning that plots look different from those produced in conventional flow. To ensure that clean populations were analyzed, conservative gating was used to identify our B cell populations. We found that the IgD^–^CD27^+^ population had a significantly higher proportion of IgM and IgA cells than the IgD^–^CD27^–^ population (*P* = 0.04 and *P* = 0.008, respectively). The proportion of IgM^+^ and IgA^+^ cells together increased further with age in both populations (Fig. 1B).

### Immunoglobulin IGHV gene repertoire of IgM^+^ B cells

**Figure 2:**
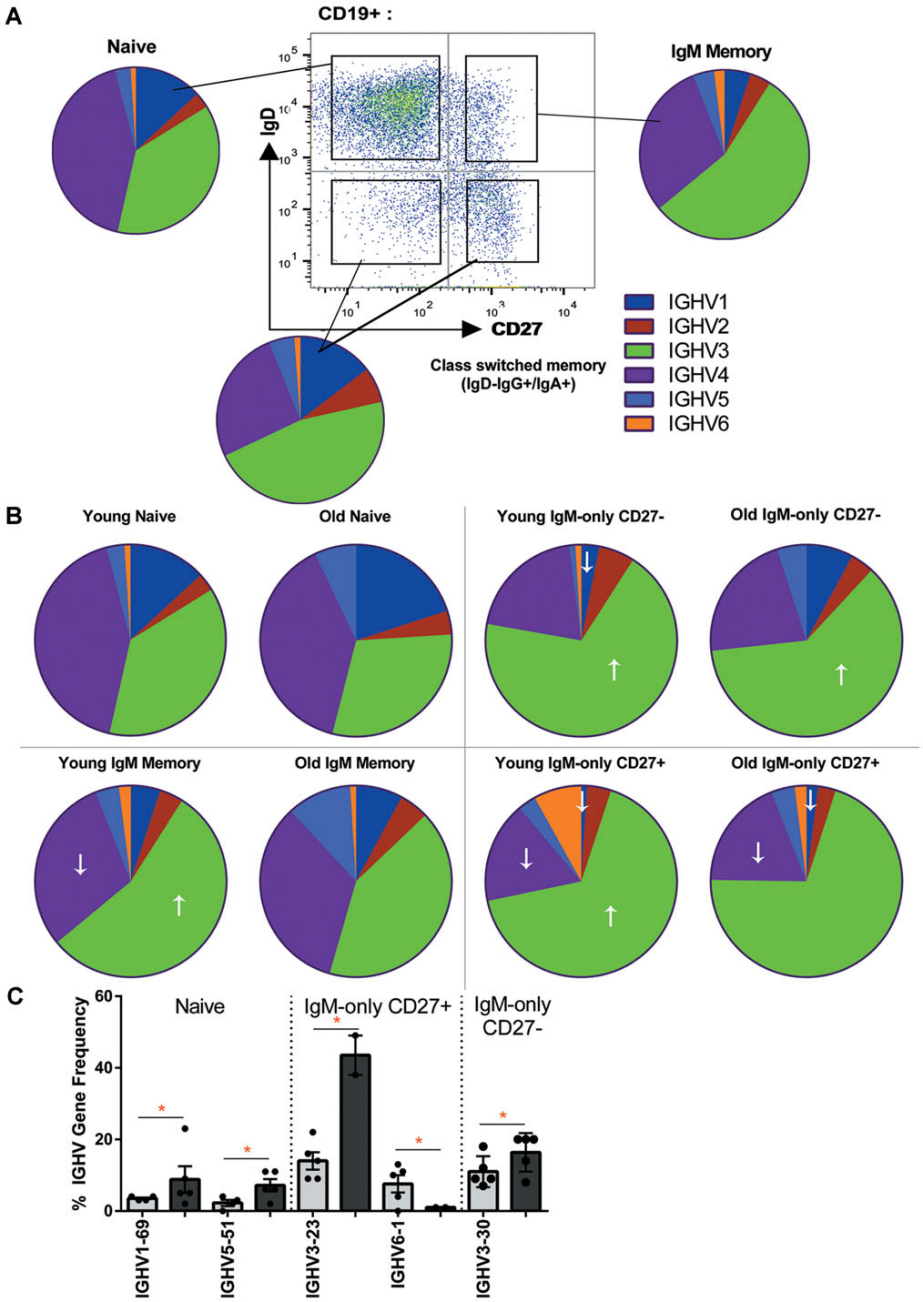
Immunoglobulin *IGHV* gene repertoire of IgM^+^ B cells. (A) *IGHV* family usage within naive, IgM memory, and class switched (IgD^–^IgG^+^/IgA^+^) cells as identified using conventional flow cytometry. (B) Average *IGHV* family usage within naive, IgM memory, IgM‐only (IgD^–^) CD27^–^, and IgM‐only (IgD^–^) CD27^+^ cells from young and old donors. ↑ indicates a significant increase in that *IGHV* gene family usage compared to the frequency in naive cells. ↓ indicates a significant decrease in that *IGHV* gene family usage compared to the frequency in naive cells. (C) Frequency usage of individual *IGHV* genes in different IgM^+^ cell types from young (light gray) and old (dark gray) donors. *n* = 8 young (light gray) and *n* = 8 old donors (dark gray). Significance was determined by 2‐way ANOVA with Sidak or Tukey post hoc testing and correction for multiple comparisons

To determine whether these IgM‐only cells were related to IgM memory cells, we compared the repertoires of four different IgM^+^ cell types (naive, IgM memory, IgD**^–^**CD27^–^, IgD^–^CD27^+^) using conventional flow cytometry. An example of the gating strategy is shown in Figure 2(A). Subsequently, the Ig genes were amplified using IgM‐specific constant‐region primers, to identify IgM^+^ sequences. After quality control to ensure only full‐length VH to Cμ sequences were represented, a total of 71,681 IgM sequences were obtained from six young and eight old individuals (Table 1).  After heavy chain complementarity determining region 3 (*CDR‐H3*) clustering to identify related sequences, we identified 31,928 unique *VDJ* gene rearrangements (Table 1). From this point onward, only unique VDJ rearrangements were used in the analysis.

**Table 1:** IgM sequences from four different IgM^+^ cell types

	Naive	IgM memory	IgD^−^CD27^+^	IgD^−^CD27^+^
Young				
Total IgM sequences	2110	17,200	4425	4969
Unique IgM rearrangements	1386	9302	2142	773
Old				
Total IgM sequences	8027	25,820	7586	1544
Unique IgM rearrangements	4398	10,176	3321	430

Our previous work showed that the *IGHV* family usage of IgM memory cells differs from that of both naive cells and switched memory cells (IgD^–^IgA^+^/IgG^+^) (Fig. 2A).  We used the sorting strategy in Figure 2(A) and we analyzed the *IGHV* gene use in our samples and found that, unlike the switched cells, *IGHV1* usage is significantly reduced in IgM‐only CD27^+^ and IgM‐only CD27^–^ populations compared to naive cells (Fig. 2B, *P* = 0.0092 and 0.0431, respectively). *IGHV3* is significantly higher in all IgM populations than in young naive cells (*P* < 0.0001 for all comparisons). This is at the expense of *IGHV1* and *IGHV4 (P* < 0.0001, < 0.0001, and 0.0034 for *IGHV4* IgM‐only CD27^+^, IgM‐only CD27^–^, and IgM memory).  However, with increasing age, the naive and IgM memory repertoires change slightly, such that there is no longer a significant difference between the two in their *IGHV3* use (Fig. 2B, *P* = 0.1929) and there is significantly lower *IGHV3* in IgM memory cells in the old (Fig. 2B, 54.5% in the young, 41.4% in the old, *P* = 0.0085). The IgM‐only cells retain their distinctive *IGHV1*, *3*, and *4* family use with age.  An analysis of individual *IGHV* genes showed very few significant differences with age.  In naive cells, there was an increase in the usage of *IGHV1‐69* and *IGHV5‐51*, in IgM‐only CD27^+^ cells, there was an increase in *IGHV3‐23* and a small reduction in *IGHV6‐1*, and in IgM‐only CD27^–^ cells an increase in *IGHV3‐30* (Fig. 2C). Therefore, it appears that age‐related changes affect selection at the family level but not at the individual IGHV gene level.

### CDR‐H3 characteristics within different IgM^+^ B cells

**Figure 3:**
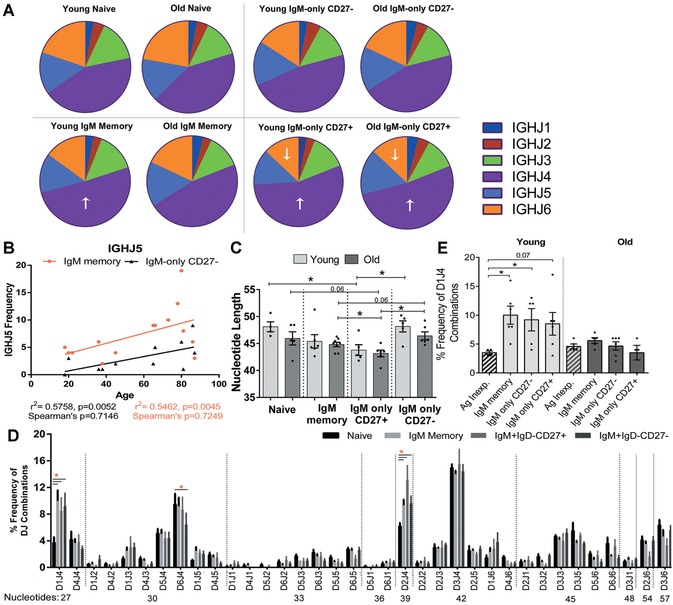
CDR‐H3 characteristics within different IgM^+^ B cells. (A) Average *IGHJ* family usage within naive, IgM memory, IgM‐only (IgD^–^) CD27^–^, and IgM‐only (IgD^–^) CD27^+^ cells from young and old donors. ↑ indicates a significant increase in *IGHJ* gene family usage compared to the frequency in naive cells. ↓ indicates a significant decrease in *IGHJ* gene family usage compared to the frequency in naive cells. (B) Correlation between *IGHJ5* family usage and age within IgM memory cells (red squares) and IgM‐only CD27^–^ (black triangles). (C) Average CDR‐H3 length, in nucleotides, between different IgM^+^ cells from young (light gray) and old (dark gray) donors. (D) Frequency of each *IGHD* and *IGHJ* (DJ) combination within naive and all IgM^+^ memory cells (IgM memory, IgD^–^CD27^–^, and IgD^–^CD27^+^). Combinations are listed in order of increasing nucleotide length, with the nucleotide size written below the combination. (E) Frequency usage of the *IGHD1* and *IGHJ4* (D1J4) gene combination within different IgM^+^ memory B cells (plain bars) compared to naive cells (diagonal lines) within young (light gray) and old (dark gray) donors. *n* = 8 young (light gray) and *n* = 8 old donors (dark gray). Significance was determined by 2‐way ANOVA with Tukey *post hoc* testing and correction for multiple comparisons; bars are SEM. Spearman's statistic was determined for correlations where stated.

We have previously shown that there are general characteristics of the CDR‐H3 region, such as the overall size and hydrophobicity that predominate in memory B cell populations compared to naive cells.^9^, ^15^ The size of the CDR‐H3 region is affected by *IGHJ* use, and, since *IGHJ6* is larger than the other *IGHJ* genes, it is quite often associated with a larger CDR‐H3 region.  In common with previous findings, we see an increase of *IGHJ4* in CD27^+^ cells compared to naive cells (Fig. 3A, *P* < 0.0001 and *P* = 0.0012 for IgM‐only CD27^+^ and IgM memory cells, respectively).  This is often at the expense of *IGHJ6 (P* = 0.0123 IgM‐only CD27^+^). CD27^+^ IgM‐only cells have a greater skew toward increased *IGHJ4* and decreased *IGHJ6* than do IgM memory cells (Fig. 3A). The increase in *IGHJ4* in IgM memory cells from the young is not observed in the old, thus showing a further age‐related change, in addition to *IGHV* usage, in the repertoire of IgM memory cells (young 43.8–51.3%, *P* = 0.0026; old 42.4–46.8%, *P* = 0.1717). There was a small but statistically significant increase of *IGHJ5* use in both older IgM memory and CD27^–^ IgM‐only populations (Fig. 3B). Generally, the picture of *IGHJ* usage shows that the IgM memory population has more in common with the IgM‐only CD27^–^ population than with the IgM‐only CD27^+^ population. In contrast to this observation, the size of CDR‐H3 decreases in both the IgM memory and the IgM‐only CD27^+^ populations compared to naive cells, but there is no decreased CDR‐H3 size in the IgM‐only CD27^–^ population (Fig. 3C).  The size of the CDR‐H3 region is also affected by the *IGHD* use, and in theory a number of different *IGHD–IGHJ* combinations can produce the same size of CDR‐H3 (Fig. 3D).  Nevertheless, the other qualities of the CDR‐H3 region are important, as evidenced by the fact that different *IGHD–IGHJ* combinations that form fragments of the same size are used to different extents in the repertoire.  For example, within fragment size 30, the combinations *IGHD5–IGHJ4* and *IGHD6–IGHJ4* are used more frequently than *IGHD1–IGHJ2* or *IGHD4–IGHJ2*, despite them all forming the same size fragments. Additionally, a general qualitative positive selection is shown, whereby particular combinations can be increased or decreased in memory cells compared to the naive population (Fig. 3D).  Of particular interest, there is a significantly increased use of the *IGHD1–IGHJ4* combination in memory cells, which is much less evident in the older samples than in the young (Fig. 3E).

### Heterogeneity of IgM‐expressing memory cells

The above data would indicate that the three types of IgM^+^ memory cell are distinct from each other.  We also highlight some age‐related differences.  One of the issues in looking at repertoire characteristics is that if one looks at a mixed population of cells the observations are averaged values over any different populations in the mix.  Since the origin of IgM memory cells has been questioned, it is quite possible that there are different subpopulations of IgM memory cells. We have shown that IgM memory cells vary in their CD24 and CD38 expression.^21^  If distinct subpopulations change differentially with age, that could also explain some age‐related changes in repertoire.  In addition to the finding of a B1‐like population in IgM memory cells,^12^ a recent paper has shown that IL‐10–producing regulatory B cells are also present.^22^

**Figure 4:**
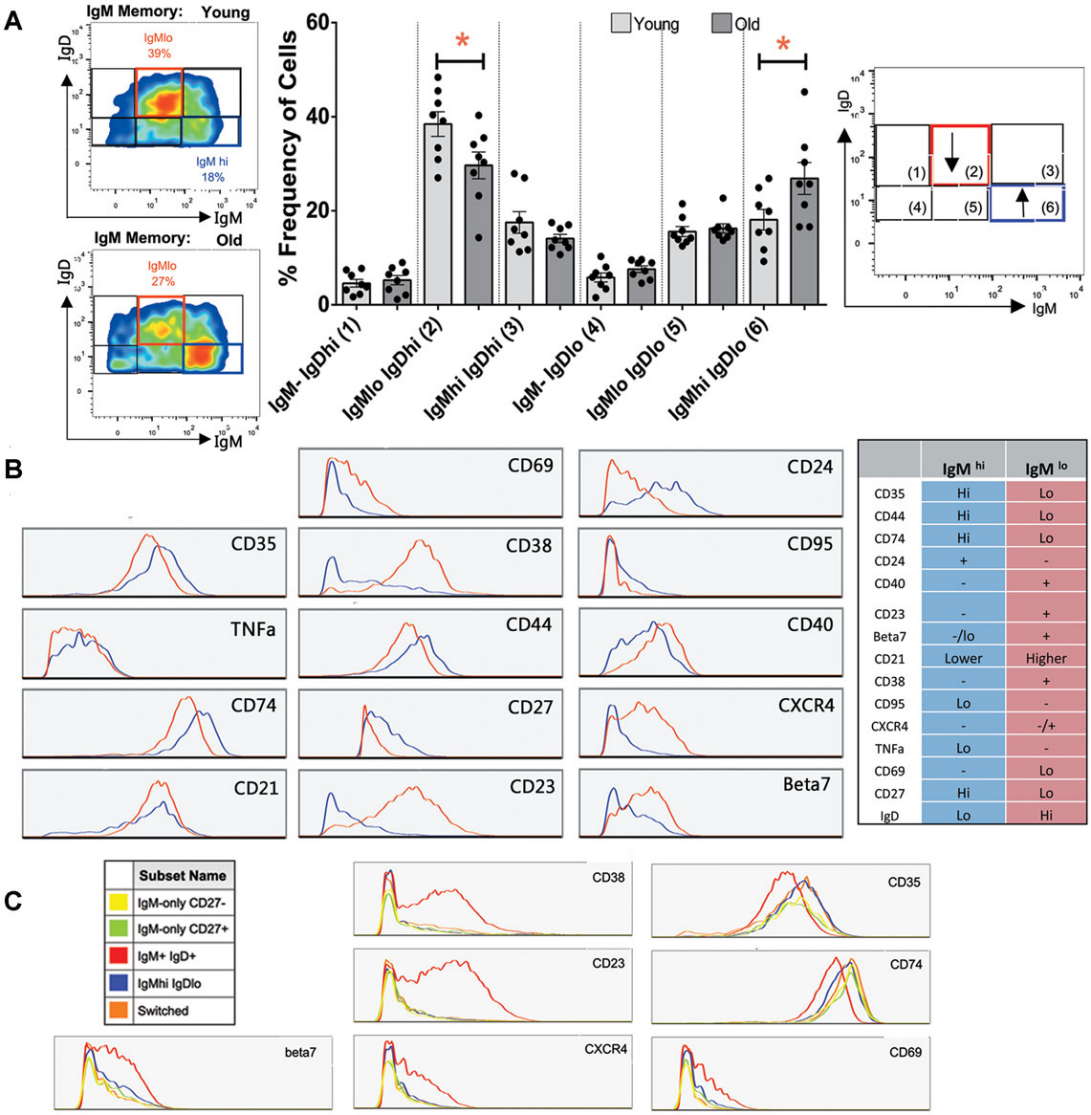
IgM memory cell heterogeneity: IgM and IgD expression using mass cytometry. (A) Pregated IgM memory cells (IgD^+^CD27^+^) were further split on the basis of expression of IgD and IgM into six gates. The IgM^lo^IgD^hi^ (IgM^lo^) population is shown within the red box and the IgM^hi^IgD^lo^ (IgD^hi^) population within the blue box. Representative plots from young and old individuals are shown. (B) Quantification of the frequency of each IgM/IgD population within young (light gray) and old (dark gray) groups, with a schematic indicating significant age‐related changes with arrows. Expression histograms for IgM^hi^ and IgM^lo^ cells were overlaid for comparison between populations. Representative histograms are from a single donor, and the phenotype of each population is summarized to the right. TNFα and CD74 are intracellular‐staining and all others are surface‐staining markers. Statistical significance was determined using paired *t*‐tests (*P* < 0.05) to compare either the mean signal between cell types (CD35 and CD74) or comparing the frequency of positive cells in each population. (C) Expression histograms overlaid to compare IgM^lo^IgD^hi^ (IgM^lo^,red) and IgM^hi^IgD^lo^ (IgD^hi^, blue) cells to switched cells (IgD^–^IgG^+^/IgA^+^, orange), IgM‐only CD27^+^ (green), and IgM‐only CD27^–^ cells (yellow).

Although we call the IgD^+^CD27^+^ B cells IgM memory cells, it was suspected that further heterogeneity within IgM memory cells existed. Using a panel of 30 different metal‐tagged antibodies and undertaking mass cytometry, we saw that the levels of IgM expression are quite varied.  In particular, we found two subpopulations of cells that were distinguishable by variation in IgD and IgM level, and these changed with age.  An IgM^lo^IgD^hi^ population decreased from 39% to 29% with age, while an IgM^hi^IgD^lo^ population increased with age from 18% to 27% (Fig. 4A).  These two populations were distinguishable by other markers, such as CD23, CD24, CD38, CXCR4, β7, and CD40 (Fig. 4B).  The IgM^hi^IgD^lo^ population has more surface markers in common with both IgM‐only and switched memory cells than with IgM^lo^IgD^hi^ B cells (Fig. 4C).

## Discussion

We have shown that all antigen‐experienced IgM^+^ cells differ in repertoire from naive cells, but there are also subtle differences between the different types of IgM^+^ cells. IgM‐only cells appear to increase with age (Fig. 1), which may be a reflection of the decreased capacity for class switching that has been previously reported.^23^ The IgM memory cells have a different *IGHV* repertoire from the IgM‐only populations, while their *IGHJ* repertoire is similar to that in CD27^–^ IgM‐only, but not that of CD27^+^ IgM‐only cells.  The repertoire of IgM memory cells appears to be most affected by age, with the characteristic increase in *IGHV3/IGHJ4* family usage and decrease in *IGHV4* usage not being seen in older patients (Figs. 2B and 3A), suggesting a possible change in gene family selection with age.

The CD27^–^ IgM‐only cells are distinct from the other antigen‐experienced IgM^+^ cells as they do not show the decrease in CDR‐H3 size that we normally see between naive and memory repertoires. All of our previous observations regarding CDR‐H3 size in the repertoire indicate that small CDR‐H3 size is a feature of antigen‐selected memory cells.^14^, ^15^, ^24^ It is also well accepted that hypermutation is a feature of memory cells, yet CD27^–^IgD^–^ memory cells have fewer mutations and thus also differ from CD27^+^IgD^–^ memory cells in this respect, having fewer mutations.^9^, ^15^, ^25^ It has been hypothesized that the CD27^–^IgD^–^ memory cells are exhausted memory cells. If this were the case, we would not expect differences in CDR‐H3 use or hypermutation from CD27^+^ memory cells. Rather, we postulate that these cells may have been initially activated in an immune response, but could then have perhaps undergone a tolerance event, with a downregulation of activation markers and possibly reduced participation in the immune response. The finding of increased numbers of these cells in association with aging and autoimmune diseases would be in agreement with this hypothesis.

This work has also highlighted the fact that, although subtle repertoire differences in memory cells can be seen with age, they may be a reflection of differences that are present in naive cells in the first instance. Although we do show a change in selection of *IGHD1–IGHJ4* in the memory repertoire (Fig. 3E), it is also clear that naive cells may be different in the older population (Fig. 2B). It may also be the case that the populations we have studied are heterogeneous, and that changes in the relative proportions of any composite subpopulations could result in overall repertoire changes. We have illustrated IgM memory heterogeneity and an age‐related change in subpopulation frequency.

IgM memory cells are of particular interest owing to their importance in protection against T‐independent antigens, such as pneumococcal antigens, and the susceptibility of older people to pneumococcal disease. We have shown evidence to support the hypothesis that they are a mixed population, and that the repertoire changes we see here may well be muted as a result of averaging across different populations.  Further work to separate out the IgM^hi^ subpopulation for repertoire analysis would be required to determine whether this population more closely matched the IgM‐only cells, but the phenotypic analysis presented here indicate that they might. Since IgM‐only cells are T dependent (being absent in CD40‐deficient hyper‐IgM syndrome),^4^ we could hypothesize that the other major subpopulation of IgM memory cells, with low expression of IgM and high expression of IgD, might be the population thought to be responsible for polysaccharide T‐independent responses. Thus, not only has the population of IgM memory cells as a whole decreased, but also within this population there are further key changes that could significantly affect the older immune system with respect to T‐independent responses. B_reg_ cells have been reported to be enriched within the IgM memory population.^22^  However, we do not know where they would fall in our two subpopulations.  Total IL‐10^+^ B_reg_ cells have been shown to have a high expression of IgM;^22^ therefore, we could hypothesize that they may be found within the IgM^hi^ population.
